# KRAS Binders Hidden in Nature

**DOI:** 10.1002/chem.201902810

**Published:** 2019-07-25

**Authors:** Andreas Bergner, Xiaoling Cockcroft, Gerhard Fischer, Andreas Gollner, Wolfgang Hela, Roland Kousek, Andreas Mantoulidis, Laetitia J. Martin, Moriz Mayer, Barbara Müllauer, Gabriella Siszler, Bernhard Wolkerstorfer, Dirk Kessler, Darryl B. McConnell

**Affiliations:** ^1^ Boehringer Ingelheim RCV GmbH & Co KG Doktor-Boehringer-Gasse 5-11 1120 Vienna Austria; ^2^ Present address: F. Hoffmann-La Roche Ltd. Grenzacherstrasse 124 4070 Basel Switzerland

**Keywords:** KRAS, natural products, NMR spectroscopy, virtual screening, X-ray crystallography

## Abstract

Natural products have proven to be a rich source of molecular architectures for drugs. Here, an integrated approach to natural product screening is proposed, which uncovered eight new natural product scaffolds for KRAS—the most frequently mutated oncogenic driver in human cancers, which has remained thus far undrugged. The approach combines aspects of virtual screening, fragment‐based screening, structure‐activity relationships (SAR) by NMR, and structure‐based drug discovery to overcome the limitations in traditional natural product approaches. By using our approach, a new “snugness of fit” scoring function and the first crystal‐soaking system of the active form of KRAS^G12D^, the protein–ligand X‐ray structures of a tricyclic indolopyrrole fungal alkaloid and an indoloisoquinolinone have been successfully elucidated. The natural product KRAS hits discovered provide fruitful ground for the optimization of highly potent natural‐product‐based inhibitors of the active form of oncogenic RAS. This integrated approach for screening natural products also holds promise for other “undruggable” targets.

Natural products have proven to be a rich source of molecular architectures which bind to drug targets.^1^ In contrast to synthetic small molecules, these secondary metabolites, which have evolved to protect host organisms, tend to feature more stereogenic centers, contain rigid cores and multiple ring systems with low degrees of conformational entropy (compounds **1**–**6**, Figure 1).^2^ However, the future role of natural products in drug discovery is constrained due to the limitations in isolation of pure natural products for screening, hit validation and hit characterization.^3^ KRAS is the most frequently mutated oncogene and belongs to the protein family of small GTPases that function as binary molecular switches involved in cell signaling.^4^ Although KRAS could serve as an excellent target for many cancers, no therapeutic agent directly targeting RAS has been clinically approved. The main reason for this is the lack of druggable pockets on the surface of RAS. In recent years, the existence of two pockets on the surface of KRAS has been discovered, which could potentially be amenable to small‐molecule drug discovery.^5^ The shallow, polar pocket situated between the switch I and switch II region of KRAS (SI/II‐pocket) is of particular interest because it is present in the active, oncogenic form of KRAS. However, molecular scaffolds capable of binding to the active form of KRAS remain scarce and are of weak affinity.^6^ In this paper, we describe an integrated approach to screening natural products in which virtual screening of natural products has been combined with elements of fragment‐based screening,^7^ the use of SAR by NMR,^8^ and structure‐based drug discovery^9^ to uncover natural‐product‐based KRAS binders.

**Figure 1:**
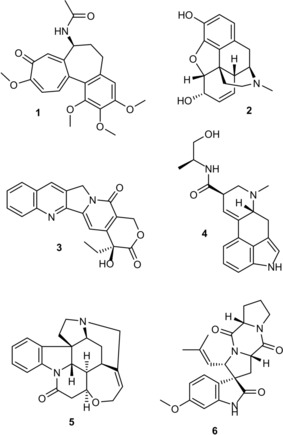
Secondary metabolites with multiple fused ring architectures including the tricyclic cycloheptatrienone colchicine (**1**), the tetrahydroisoquinoline derived pentacycle morphine (**2**), the pentacyclic quinolone camptothecin (**3**), the tetracyclic indole ergonovine (**4**), the heptacyclic indoline strychnine (**5**) and the spiroindolinone spyrotryprostatin A (**6**).

Our previous attempts to identify starting points through high‐throughput screening of 1.6 million compounds, including a diversity‐oriented synthesis library (150 000 compounds), failed to deliver any inhibitors of GTP‐KRAS. This demonstrates that the chemical space covered by our historical compound collection was not sufficiently complementary to the pockets on KRAS in the affinity range tested by biochemical and cellular assays. Natural products, however, could represent an area of chemical space containing molecular architectures with sufficient complementarity to KRAS.

Considering that many natural product structures are not ideal for virtual screening due to their large size and high degrees of substitution, a chemoinformatics based PipelinePilot workflow, analogous to that used by other groups,^10^ deconstructed all structures in the CRC *Dictionary of Natural Products* (DNP)^11^ (180 000 entries at the time of application). This produced a library of 226 000 original and deconstructed natural products amenable to virtual screening, referred to subsequently as the virtual DNP (vDNP). At the time of the virtual screening campaign, only one X‐ray co‐crystal structure of a ligand bound to the active form of KRAS had been reported.5b Due to ambiguity in the electron density for the proposed binding mode in this structure, we decided instead to use the published GDP‐KRAS structure (PDB code 4EPV) with indole **7** bound as published by the Fesik group,5a as a surrogate for GTP‐KRAS.

The vDNP was docked by using Glide into the SI/II‐pocket of GDP‐KRAS. The compounds with the best 500 molecular‐weight‐normalized docking scores^12^ (Glide score/MW) were visually inspected, and compounds that mimicked the known indole occupation of the SI/II‐pocket (Figure 3 A) and formed H‐bonds with D54 were selected. Compounds deemed chemically unstable were removed. 3‐Substituted indoles were also not pursued further because they did not provide sufficient structural novelty as a starting point versus **7** (Figure 2).

**Figure 2:**
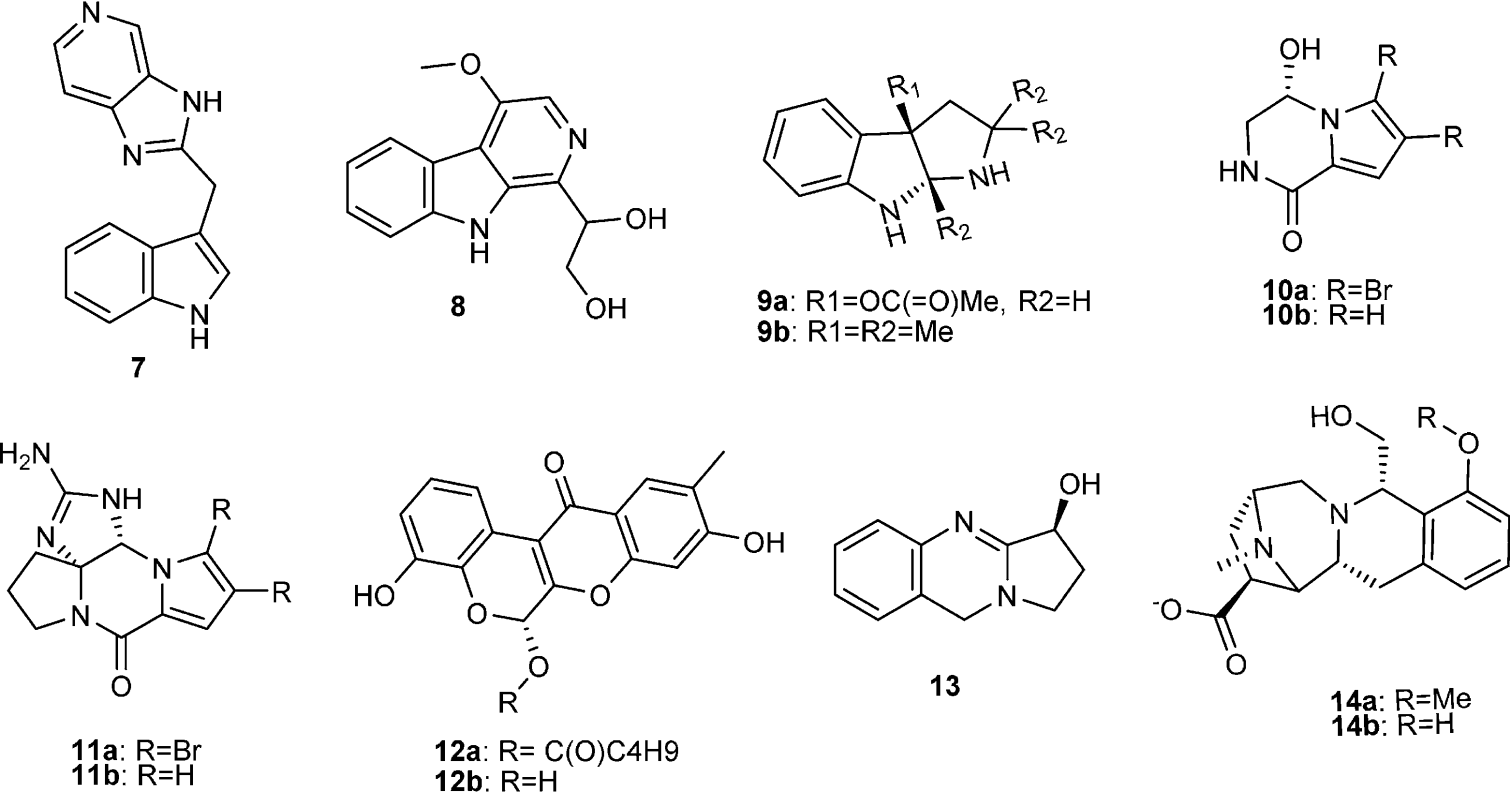
Selected natural product virtual KRAS hits and indole **7** present in the GDP‐KRAS co‐crystal structure (PDB code 4EPV) used for docking.

From the virtual screen of the vDNP, natural product hits isolated from fungi, sponges, plants and bacteria were identified (Figure 2). β‐Carbolines and γ‐carbolines, which are relatively ubiquitous in nature, substituted at the 1‐, 2‐ and 3‐positions were found. β‐Carboline **8** (Figure 2), similar to indole **7**, filled the lipophilic SI/II‐pocket flanked by L56 and K5 and formed two H‐bonds to D54: one through the indole NH and a second through the glycerol substituent (Figure 3 B). The tricyclic indolopyrrole alkaloid **9 a** (Figure 2) first isolated from the fungus *Fusarium incarnatum* mimics the indole binding mode of **7** and **8**, while forming a second H‐bond to D54 through the pyrrole nitrogen (Figure 3 C). The marine sponges *Agelas longissima* and *Phakellia flabellate* provided two KRAS virtual hits derived from bromopyrrole alkaloids, namely, the debrominated analogue **10 b** of longamide (**10 a**) (Figure 2) and the tetracyclic guanidine scaffold Phakellin **11 b**, which co‐occurs with its dibromo analogue **11 a** ((+)‐dibromophakellin; Figure 2). Compound **10 b** forms one H‐bond to D54 through a hydroxyl group (Figure 3 d), whereas **11 b** forms two H‐bonds to D54 through the guanidine group (Figure 3 e). Two virtual hits were also uncovered from plant secondary metabolites, namely, diffusarotenoid **12 a** and vasicine **13**, a tricyclic quinazoline (Figure 2). The *cis*‐fused tetrahydrochromenochromene ring system of the de‐esterified diffusarotenoid **12 b** (Figure 2) complemented the shape of the SI/II‐pocket while forming two H‐bonds to D54: one through the phenolic oxygen and the backbone carbonyl, and one between the 6‐hydroxyl and the side chain (Figure 3 f). Compound **13** also occupies the SI/II‐pocket and forms an H‐bond with D54 through the 3‐hydroxy group (Figure 3 g). Deconstruction of the methyl ether of quinocarcinol **14 a,** isolated from the bacteria Streptomyces melanovinaceus, led to the virtual hit **14 b** (Figure 2) for which the iminoazepinoisoquinoline skeleton occupies the SI/II‐pocket and forms two H‐bonds to the side chain of D54 with the phenolic and alcoholic oxygens (Figure 3 h).

**Figure 3:**
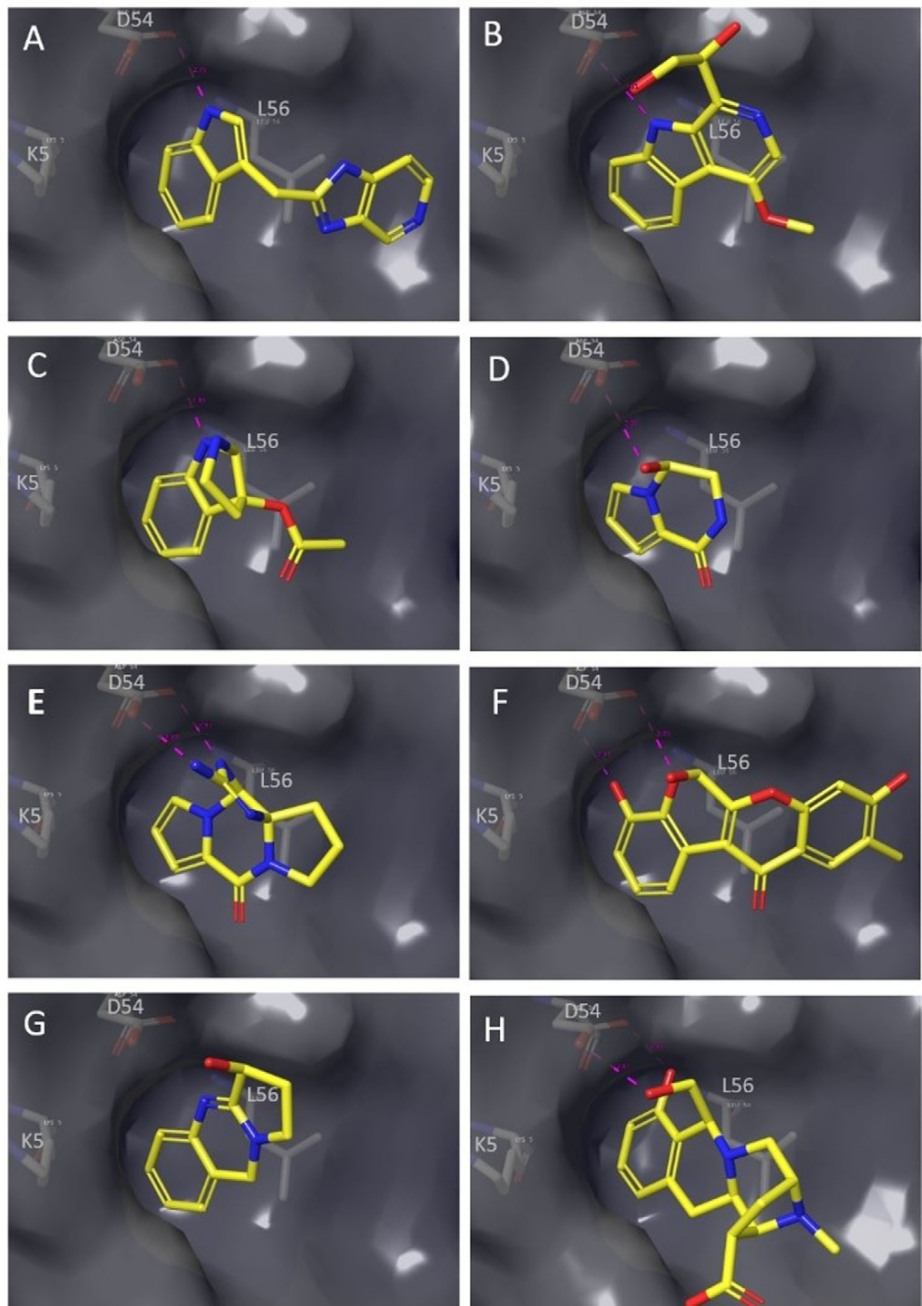
Docking poses of virtual hits in the switch I/II‐pocket. (A) **7**, (B) **8**, (C) **9 a**, (D) **10 b**, (E) **11 b**, (F) **12 b**, (G) **13**, (H) **14 b**.

To validate the virtual screen, **9 b**, a close analogue of the tricyclic indolopyrrole alkaloid **9 a** was tested for binding to GCP‐KRAS by using NMR. Compound **9 b** demonstrated cross peak shifts in the 2D ^1^H/^15^N HSQC NMR spectra of GCP‐KRAS^G12D^ (Figure 4 a), confirming that these virtual hits do indeed bind to the active form of KRAS. The dissociation constant (*K*_D_) for **9 b**, as measured by NMR, was in the millimolar range with no saturation obtained with the concentrations tested (up to 1.5 mm, Figure 4 b). To be able to perform high throughput crystallization of very weakly binding fragments (*K*_D_>1 mm) with KRAS, such as those reported here, we developed a robust crystal‐soaking system for active KRAS^G12D^. By using GDP‐KRAS^G12D^, we exchanged the nucleotide with the non‐hydrolysable GTP analogue GMP‐PCP (GCP) to yield a novel crystal form amenable to high‐throughput crystallization, which mimics the beta sheet dimeric form of active KRASG12D^13^ (Figure 4 c). With this soaking system, we were able to determine the co‐crystal structure of **9 b** with GCP‐KRAS at a resolution of 1.2 Å (Figure 4 c). The observed binding mode confirmed the docking prediction (Figure 4 d), namely binding to the SI/II‐pocket and the formation of two H‐bonds to D54.

**Figure 4:**
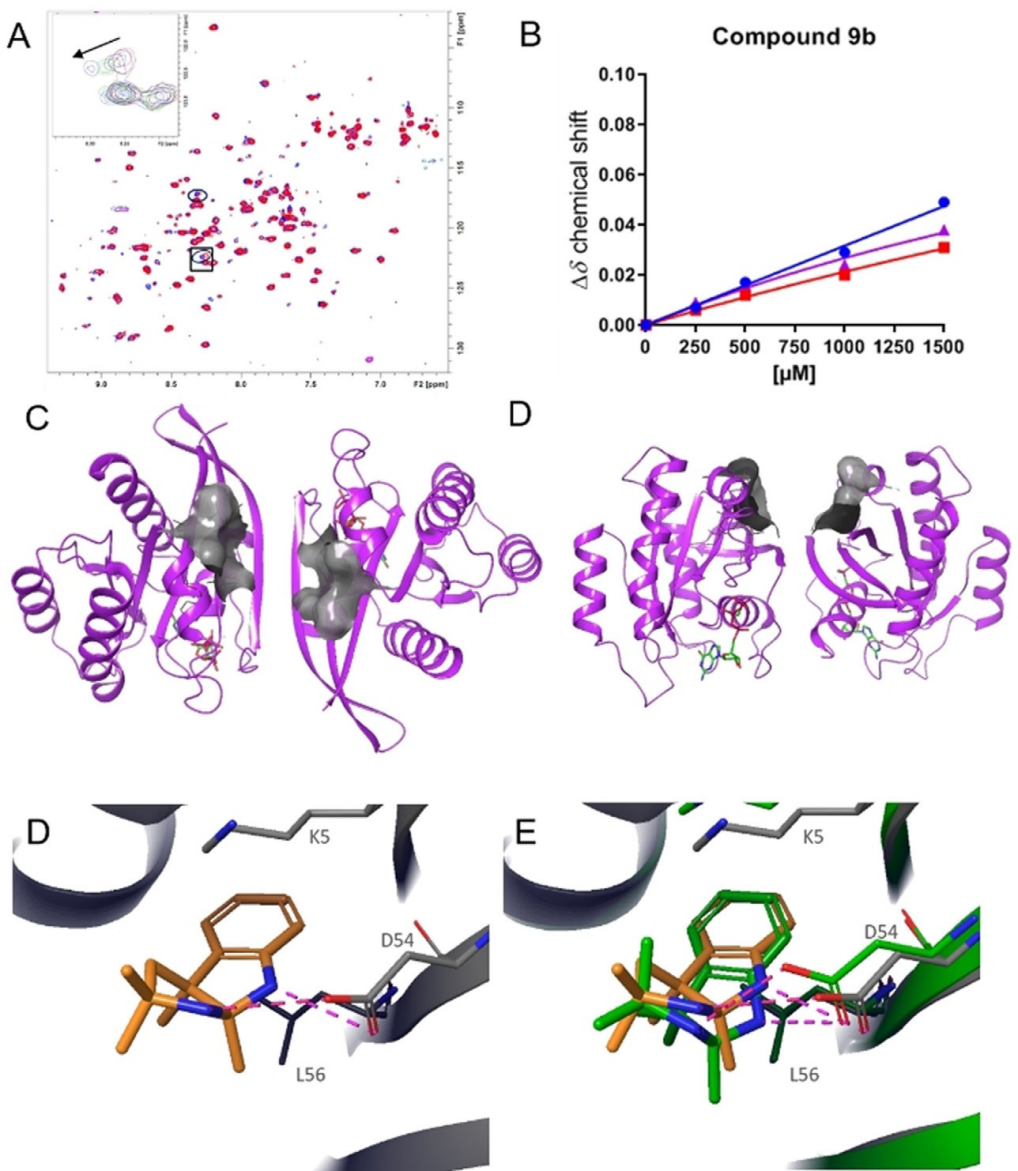
NMR and X‐ray crystal structure of indolopyrrole **9 b**. (A) Dose‐dependent cross peak shifts in the 2D ^1^H/^15^N HSQC NMR spectra of GCP‐KRAS^G12D^ on addition of **9 b**. (B) NMR *K*_D_ titration of **9 b** binding to GCP‐KRAS^G12D^. (C) Top view and (D) side view of the high‐throughput soaking crystallization system for KRAS^G12D^ with the SI/II‐pocket surface depicted in grey. (E) X‐Ray co‐crystal structure of **9 b** binding to the SI/II‐pocket of GCP‐KRAS^G12D^. (F) X‐Ray co‐crystal structure of **9 b** binding to the SI/II‐pocket of GCP‐KRAS^G12D^ in yellow overlaid with the docking pose in green.

The docking pose of rotenoid **12 b** displayed striking complementarity to the shape of the SI/II‐pocket (Figure S2 A, Supporting Information); however, semiempirical quantum mechanics calculations (AM1, PM3, MNDO) and crystal structure analysis of structurally related compounds in the Cambridge Structural Database suggest that rotenoids exist in a flat conformation. The complementary “kinked” docking pose of **12 b** in the SI/II‐pocket of KRAS encouraged us to generate a sub‐library of 1383 rotenoid‐like “kinked” molecules with contiguous fused rings from our corporate database for a second virtual screening campaign.

To ensure that the shape complementarity between the rigid three‐dimensional topology of our “kinked” library and the KRAS protein were assessed accurately, we developed a “snugness‐of‐fit” scoring function. The shapes of a protein and a ligand were calculated as envelopes around their respective atoms and were represented as 3D grids. Envelope overlaps that occurred at small atomic radii corresponded to those areas where the protein and ligand were close, that is, “snug”, whereas overlaps that only occurred at larger radii represented fewer snug areas. Addition of all envelope overlap grids (at different radii) provided a quantitative measure for the “snugness of fit” of the ligand at each point in space. The grids can be also used for visualizing the snugness of fit of a docked or crystallized compound using iso‐contour surfaces or color‐coded surfaces (Figure S2, Supporting Information).

The “kinked” library was virtually screened in an analogous procedure to that used for the vDNP, with the addition of the “snugness of fit” scoring function for pose post‐processing (Figure 5 a). After visual inspection, 7 compounds were selected for NMR testing and the racemic compound **15** showed NMR cross peak shifts. Both enantiomers **15 R** and **15 S** docked with conserved indole binding modes (Figure S3 A,B, Supporting Information); hence, the racemate **15** was separated into its two enantiomers **15 R** (2*R*,3*S*) and **15 S** (2*S*,3*R*). **15 R** displayed a *K*_D_ of 1 mm (Figure 5 B,C), as determined by the 2D ^1^H/^15^N HSQC NMR spectra to GCP‐KRAS^G12D^, whereas **15 S** bound more weakly with a dissociation constant in the millimolar range (Figure S3 C and Figure S3 D). Enantiomer **15 R** was successfully soaked into the crystallography system (Figure 5 D) with the crystal structure confirming the docking pose (Figure 5 E). **15 R** binds to the SI/II‐pocket of active KRAS and forms two chelating H‐bonds to D54 with an N−O distance of 2.9 and 3.0 Å, respectively, and a weaker third H‐bond to the K9 side chain through the carbonyl group with an O−N distance of 3.4 Å.

**Figure 5:**
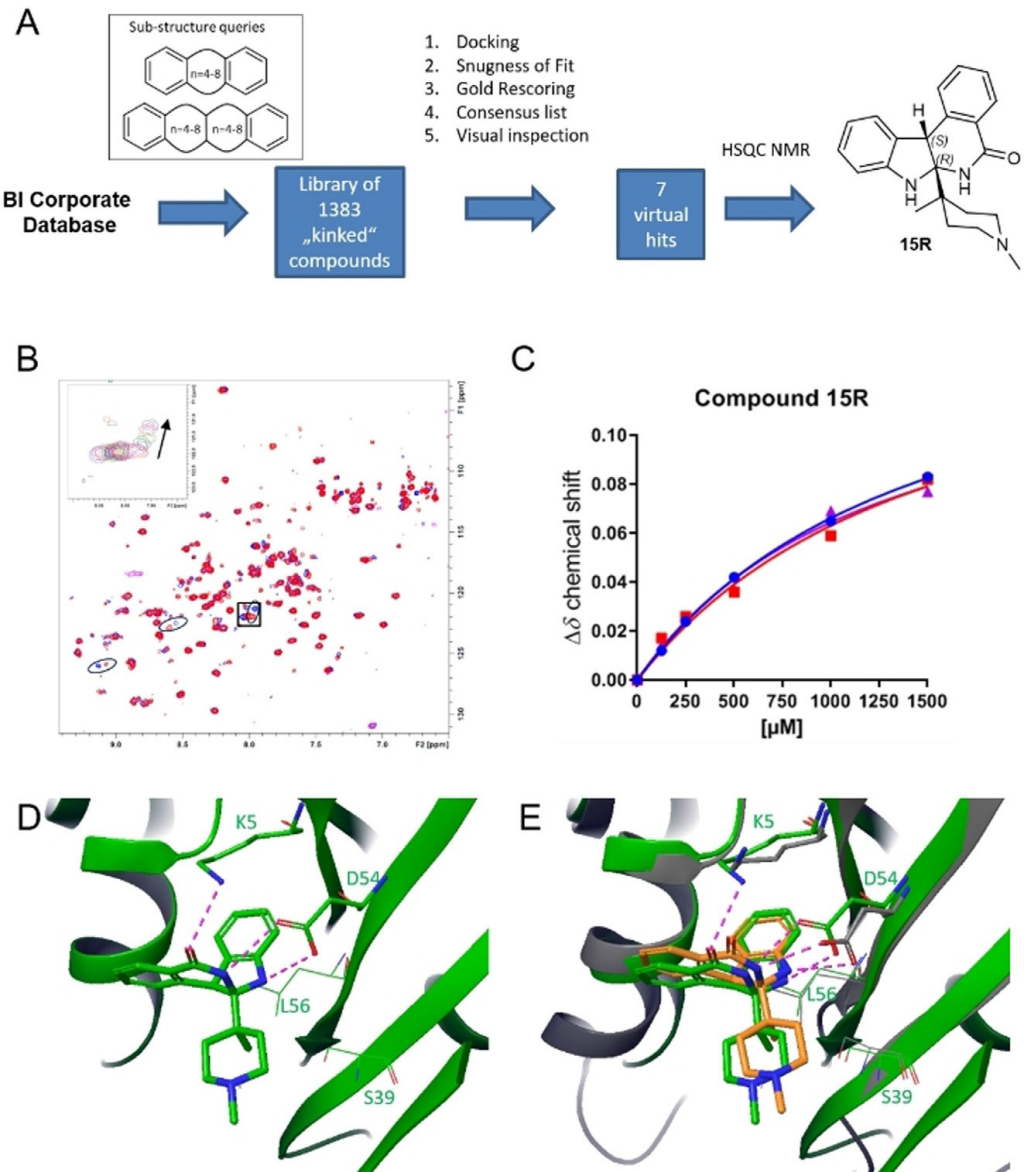
“Kinked” compound library virtual screen and crystal structure of indoloisoquinolinone **15**. (A) Schematic representation of the generation of the “kinked” compound library and virtual screen leading to the hit **15**. (B) Dose dependent cross peak shifts in the 2D ^1^H/^15^N HSQC NMR spectra of GCP‐KRAS^G12D^ on addition of **15 R**. (C) NMR *K*_D_ titration of **15 R** binding to GCP‐KRAS^G12D^ showing a *K*_D_ of 1 mm. (D) X‐Ray co‐crystal structure of **15 R** binding to the SI/II‐pocket of GCP‐KRAS^G12D^. (E) X‐Ray co‐crystal structure of **15 R** binding to the SI/II‐pocket of GCP‐KRAS^G12D^ in green overlaid with the docking pose in orange.

In conclusion, from the two virtual screens conducted, virtual hits stemming from seven classes of natural products from fungi, sponges, plants, bacteria and one natural‐product‐like class from our corporate compound collection were identified. Of these, we confirmed biophysical binding to the active form of KRAS^G12D^ for the tricyclic indolopyrrole alkaloid **9 b** and the indoloisoquinolinones **15 R** and **15 S**. Importantly, we elucidated the binding mode by determining the protein–ligand complex structures of **9 b** and **15 R** with the first X‐ray crystal‐soaking system for the active form of KRAS^G12D^. The wealth of natural product KRAS hits, discovered by using this integrated approach of natural product screening, should act as inspiration for the design of more potent and selective RAS inhibitors. This approach also promises to be applicable to other “undruggable” targets beyond KRAS, for which classical screening methods have proven to be unsuccessful.

## Conflict of interest

The authors declare no conflict of interest.

## Supporting information

As a service to our authors and readers, this journal provides supporting information supplied by the authors. Such materials are peer reviewed and may be re‐organized for online delivery, but are not copy‐edited or typeset. Technical support issues arising from supporting information (other than missing files) should be addressed to the authors.

Supplementary
