# Supplementary material for: KRAS Binders Hidden in Nature
Source: Chemistry. 2019 Jul 25;25(52):12037–41. doi: 10.1002/chem.201902810 (PMC6772028; doi:10.1002/chem.201902810)
Supplement: Supplementary file 1 — Supplementary [file CHEM-25-12037-s001.pdf]

CHEMISTRY
A European Journal

Supporting Information

KRAS Binders Hidden in Nature

Andreas Bergner,[a] Xiaoling Cockcroft,[a] Gerhard Fischer,[a] Andreas Gollner,[a] Wolfgang Hela,[a] Roland Kousek,[a] Andreas Mantoulidis,[a] Laetitia J. Martin,[a, b] Moriz Mayer,[a] Barbara Müllauer,[a] Gabriella Siszler,[a] Bernhard Wolkerstorfer,[a] Dirk Kessler,[a] and Darryl B. McConnell*[a]

chem_201902810_sm_miscellaneous_information.pdf

Supplementary Information

XRAY

Cloning and Protein Production

Briefly, KRAS-4B (amino acids 1-169) was cloned with a pET2-vector into *E.coli* BL21 (DE3) cells. The construct contained a mutation G12D and an N-terminal His_6-tag followed by a TEV-cleavage site. Cells were grown in Terrific Broth media, protein production induced for 18h at 18 °C by addition of 0.2 M IPTG, cells were harvested by centrifugation and stored at -80 °C. Cells were broken by sonication, cell debris removed by centrifugation and the protein purified by Ni-NTA-affinity chromatography. After buffer exchange into 20 mM HEPES pH 7.5, 150 mM NaCl, 5 mM $MgCl_2$ supplemented with with 1 mg GDP per 20 mg protein the His_6-tag was cleaved off with TEV-protease at 4°C, which was subsequently removed by reverse Ni-NTA affinity chromatography. The protein was further purified by size exclusion chromatography in 20 mM HEPES pH 7.5, 150 mM NaCl and concentrated to approximately 40 mg/mL. Finally, the nucleotide was exchanged from GDP to GppCp (GCP) according to Eberth et al[1].

Crystallisation and Crystallography

Crystals were obtained through hanging drop vapour diffusion by mixing 1.4 µL of protein (25 mg/mL) with 0.8 µL of reservoir (52-55% 2-methyl-2,4-pentanediol, 0.06 M MES pH=6.4). For protein-ligand complex structures, the crystals were transferred to a reservoir solution containing 10 mM of ligand in DMSO and were incubated over night. Crystals were harvested by adding 20% ethyleneglycol to the reservoir as cryoprotectant and flash-cooled in liquid nitrogen.

Diffraction data were collected at the Swiss Light Source beamlines, integrated using autoPROC[2]/XDS [3], isotropically or anisotropically scaled with Aimless[4] or Staraniso[5] respectively. Molecular replacement was performed using PHASER[6] and the resulting model refined using Phenix[7], autoBUSTER[8] and Coot[9] (see Table S1 for data collection and refinement statistics). Restraint dictionaries for ligands were calculated with GRADE (GlobalPhasing, UK) and pictures created with Pymol (Schrödinger, USA).

Table S1. Crystallographic Processing and Refinement Statistics

	GCP-KRAS	9b-GCP-KRAS	15-GCP-KRAS	15R GCP-KRAS
**PDB ID**	6QUU	6QUW	6QUX	6QUV
**Data Collection***				
**Beamline**	SLS-PXIII	SLS-PXIII	SLS-PXIII	SLS-PXIII
**Wavelength [Å]**	1.00000	1.00004	0.99987	1.00000
**Space group**	$P\ 2_1$	$P\ 2_1$	$P\ 2_1$	$P\ 2_1$
**Cell dimensions**				
***a, b, c* (Å)**	42.6, 72.3, 54.7	42.5, 72.1, 54.6	42.8, 72.7, 54.9	42.4, 72.2, 54.6
α, β, γ (°)	90, 103.4, 90	90, 103.3, 90	90, 103.7, 90	90, 103.2, 90
**Max. Resolution (Å)**	42.3 – 1.5 (1.579 – 1.477)	53.1 - 1.2 (1.342 – 1.242)	53.3 – 1.6 (1.616-1.622)	53.1 – 1.5 (1.574-1.475)
**Anisotropic ellipsoid cutoffs [Å]**	1.7 – 1.5 – 1.5**	1.5 – 1.3 – 1.2**	n/a	1.5 – 1.5 – 1.7**
Rmerge	0.059 (0.7)	0.032 (0.751)	0.053 (0.597)	0.039 (0.695)
CC 1/2	1.0 (0.6)	1.0 (0.6)	1.0 (0.7)	1.0 (0.6)
$I / \sigma I$	10.9 (1.5)	16.1 (1.4)	14.3 (1.9)	14.4 (1.5)
**Completeness (%)**	93.3 (53.3)	88.7 (69.5)	99.6 (99.7)	92.8 (51.3)
**Unique reflections**	43583	61942	41613	43902
**Redundancy**	3.4	3.4	3.4	3.3
**Refinement**				
**Resolution range (Å)**	37.1 – 1.5	35.9 – 1.2	37.3 – 1.6	37.0 – 1.5
R_{work} / R_{free}	19.3 / 20.4	19.3 / 21.0	20.4 / 22.3	19.3 / 20.8

R.m.s. deviations				
Bond lengths (Å)	0.011	0.011	0.012	0.010
Bond angles (°)	1.16	1.14	1.35	1.03
Mean/ Wilson B	26 / 19	24 / 15	24 / 21	27 / 20
Ramachandran statistics (favoured/outliers) [%]	96.7 / 0.3	96.7 / 0.0	96.7 / 0.0	97.3 / 0.0

*Values in parentheses are for highest-resolution shell. ** Data were scaled anisotropically with Staraniso. Resolution is given for the principal axes of the cutoff-ellipsoid. Subsequent data processing statistics apply to this ellipsoid.

NMR Spectroscopy

All NMR experiments were acquired on a Avance III 700MHz spectrometer equipped with a cryogenically cooled 5mm TCI probe. Processing and analysis was done with Topspin 3.1 (Bruker BioSpin). Binders were identified by manual comparison of spectra in the presence of a fragment and the respective reference spectrum, compounds were qualified as non-binders if the $\Delta\delta$ chemical shift difference was < 0.01 ppm, according to the equation shown below.

Measurement of dissociation constants K_D for the compounds synthesized. $^1H/^{15}N$ SoFast HMQC experiments were recorded in 3mm NMR tubes (170µl filling) at a protein concentration of 70-100µM. Spectra were recorded with 48 scans, 128 f1 increments and 2k data points in f2. Total acquisition time was 22 minutes. 4 – 5 titration points were performed with individual samples for each titration point made from 50mM DMSO-d6 stock solutions. Total DMSO-d6 concentration was kept constant by backfilling every sample to a total of 4% DMSO. K_Ds were calculated from the differences in chemical shift induced by the small molecules upon interacting with the protein. Average chemical shifts from the ^1H and ^{15}N dimension were calculated according to the following equation:

$\Delta\delta_{obs} = \sqrt{[\delta_H^2 + (\alpha \cdot \delta_N^2)^2]}$ with α set to 0.14

The $\Delta\delta_{obs}$ values at the respective ligand concentrations and the total concentration of protein allow to fit the K_D value of the ligand as long as it is binding under fast exchange conditions as was the case for all of the compounds tested. Usually $\Delta\delta_{obs}$ values of 3 to 5 different resonances were used and an average K_D determined. The following equation was used to determine the individual K_Ds:

$$\Delta\delta_{obs} = \Delta\delta_{max}\left[([P]_t + [L]_t + KD) - [([P]_t + [L]_t + KD)^2 - 4([P]_t[L]_t)]^{1/2}\right]/2[P]_t$$

Computational Chemistry

vDNP Virtual Screening

Prior to docking, all compounds in the vDNP library were converted from 2D to 3D using Corina[9] followed by preparation using Schrodinger's Ligprep module. The initial docking was done with Schrodinger's Glide Suite[9] 2015. Standard precision docking mode was used. The protein docking grid was derived from the PDB X-ray crystal structure of GDP-KRAS, PDB code 4EPV. The 500 top ranked compounds based on the molecular weight divided by GlideScore were selected for visual inspection and further processing.

Rotenoid-like Library Virtual Screening Method

The Rotenoid-like library was prepared and docked in the same way as for the vDNP Virtual Screening. All docking poses generated with Glide were rescored in CCDC's Gold version 2015[9]. Scoring functions used included ChemScore, PLPScore, AspScore and GoldScore which were used for a consensus scoring scheme where only compounds present in the top 10% ranks of all scoring function were further pursued. Compounds that passed the consensus scoring filter were further assessed using a Snugness-of-Fit scoring function developed at BI. The final shortlist was visually inspected and compounds were ordered and followed-up experimentally.

Snugness-of-Fit calcuation

Current scoring functions are not precise enough to estimate the binding affinity of a ligand based on its placement (docking pose) in a binding site. Scoring functions are used for ranking the poses, however, depending on the target protein the recall rates of true active compounds are often poor. Conversely, successful virtual screening campaigns with low follow-up assay throughput capacities crucially depend upon reliable ranking and high recall values. As a result, following a cascade of post-processing steps, docking poses are often finally assessed by visual inspection, as in the examination of protein-ligand interactions and the shape of the ligand complementing the binding site shape.

We have developed a snugness-of-fit scoring function that aims to quantify this shape complementary based on purely geometrical considerations, in order to complement the current scoring functions for the post-processing of docking poses. The snugness calculation for a ligand pose works as follows:

The shapes of a protein and a ligand are calculated as envelopes around their respective atoms. In technical terms, they are represented as 3D-grids comprising only 1 (inside) and 0 (outside) values. If the radii of the atoms correspond to atomic vdW radii, the envelopes correspond to molecular surfaces.

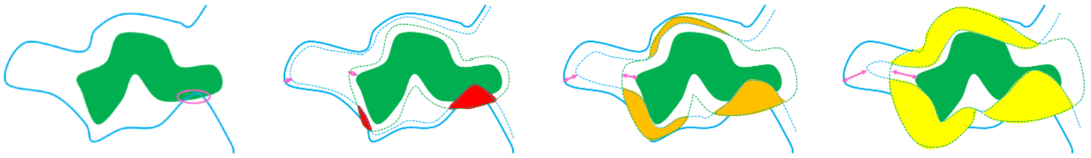

Envelopes (binary 0/1 3D-shape grids) with different atomic radii are generated for both the protein and the ligand, and the overlap between the envelopes (inside in both grids) is transferred onto another grid that then represents the overlap of protein and ligand shapes. For small radii, overlaps correspond to areas where the protein and ligand are close, i.e. “snug”. For larger radii, the envelopes inflate and hence the size of overlapping areas increases, denominating less snug areas.

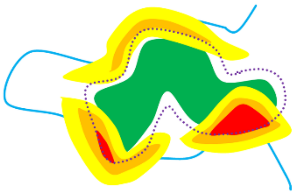

Addition of all envelope overlap-grids (with different radii) provides a quantitative measure for the snugness of fit of the ligand at each point in space. In the VS campaign, such snugness grids are calculated for each pose and used for pose ranking in the post-processing. The grids can also be used for visualizing the snugness of fit, using iso-contour surfaces or color code surfaces. The snugness grid calculation has been implemented in MOE using SVL[10].

Synthesis and Analytics

The compound 15 is part of BI’s corporate compound collection. The stereoisomeric mixture was separated to provide 15R and 15S using a chiral column on a SFC.

Analytical data for 15R:

^{1}H NMR (500 MHz, DMSO-d_6) δ 7.84 (d, *J*=7.25 Hz, 1H), 7.58-7.66 (m, 2H), 7.57 (s, 1H), 7.36-7.43 (m, 1H), 7.00 (t, *J*=7.57 Hz, 1H), 6.69 (d, *J*=7.57 Hz, 1H), 6.60-6.65 (m, 1H), 6.52-6.59 (m, 1H), 5.83 (s, 1H), 4.62 (s, 1H), 2.53-2.59 (m, 2H), 2.11 (s, 3H), 1.87-2.03 (m, 2H), 1.71-1.82 (m, 1H), 1.51-1.67 (m, 1H),

1.12-1.28 (m, 2H), 0.91 (s, 3H)^{13}C NMR (125 MHz, DMSO-d_6) δ 163.2, 148.3, 138.2, 132.4, 130.8, 127.9, 127.2, 127.1, 126.6, 123.7, 118.0, 109.6, 86.9, 50.9, 50.8, 46.0, 43.9, 31.0, 30.8, 17.4; MS: $[M+H]^+$ = 348

The chiral separation of compound 15 was carried out on a Sepiatec Prep SFC 100 system using supercritical fluid chromatography. A Chiralpak IC (250 x 20 mm, 5 µm) column with the following conditions was used for separation. Temperature 40°C, 40% Methanol + 0.1% Diethylamine, 60% CO_2, flow 60 ml/min and a backpressure of 150 bar. Compound 15R eluted first at 3.8 minutes and compound 15S second at 5.0 minutes under these conditions.

NMR experiments were recorded on Bruker Avance HD 500 MHz spectrometers equipped with a TCI cryoprobe at 298 K. Samples were dissolved in 600 µL DMSO-d6 and TMS was added as an internal standard. 1D 1H spectra were acquired with 30° excitation pulses and an interpulse delay of 4.2 s with 64k data points and 20 ppm sweep width.

1D 13C spectra were acquired with broadband composite pulse decoupling (WALTZ16) and an interpulse delay of 3.3 sec with 64 k data points and a sweep width of 240 ppm. Processing and analysis of 1D spectra was performed with Bruker Topspin 3.2 software. No zero filling was performed and spectra were manually integrated after automatic baseline correction. Chemical shifts are reported in ppm on the δ scale.

A

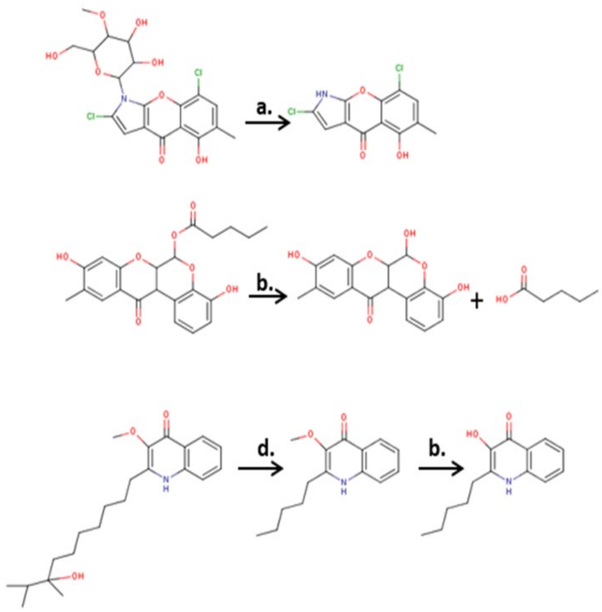

B

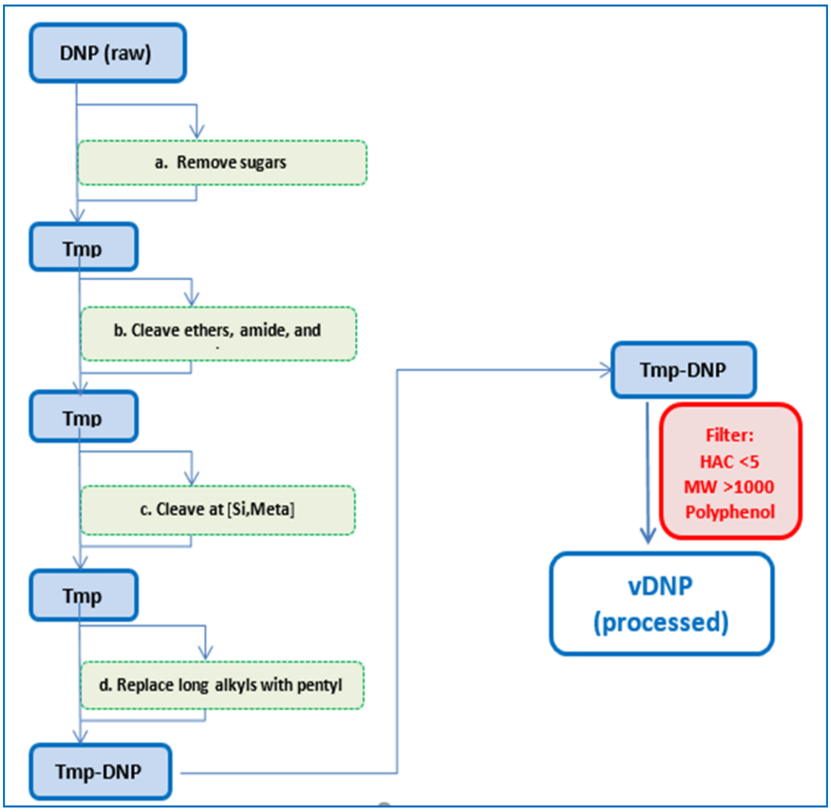

Figure S1 | Rules and chemoinformatics based DNP deconstruction workflow. (A) Examples for deconstruction rules used to generate the virtual DNP library. (b) Schematic representation of the chemoinformatics based PipelinePilot[11] workflow that deconstructed the natural products in the DNP.

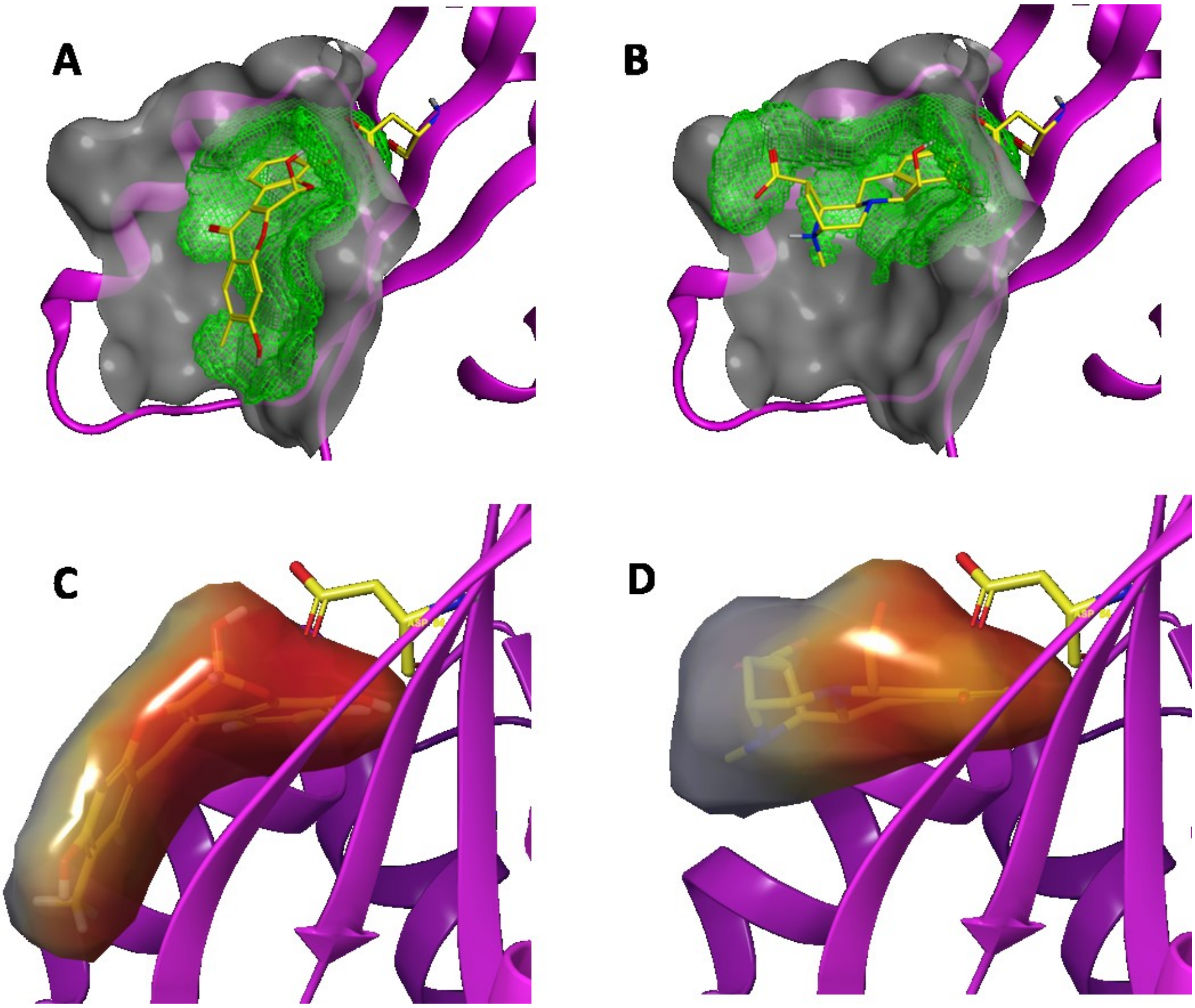

Figure S2 | Visualization of Snugness of Fit. Snugness of fit for compound **12b** (snugness ratio = 0.619, Glide score = -6.375) shown as an iso-contour surface (panel A) and as a colour-coded surface (panel C). Snuggness of fit for compound **14b** (snugness ratio = 0.573, Glide score = -6.989) shown as an iso-contour surface (panel B) and as a colour-coded surface (panel D).

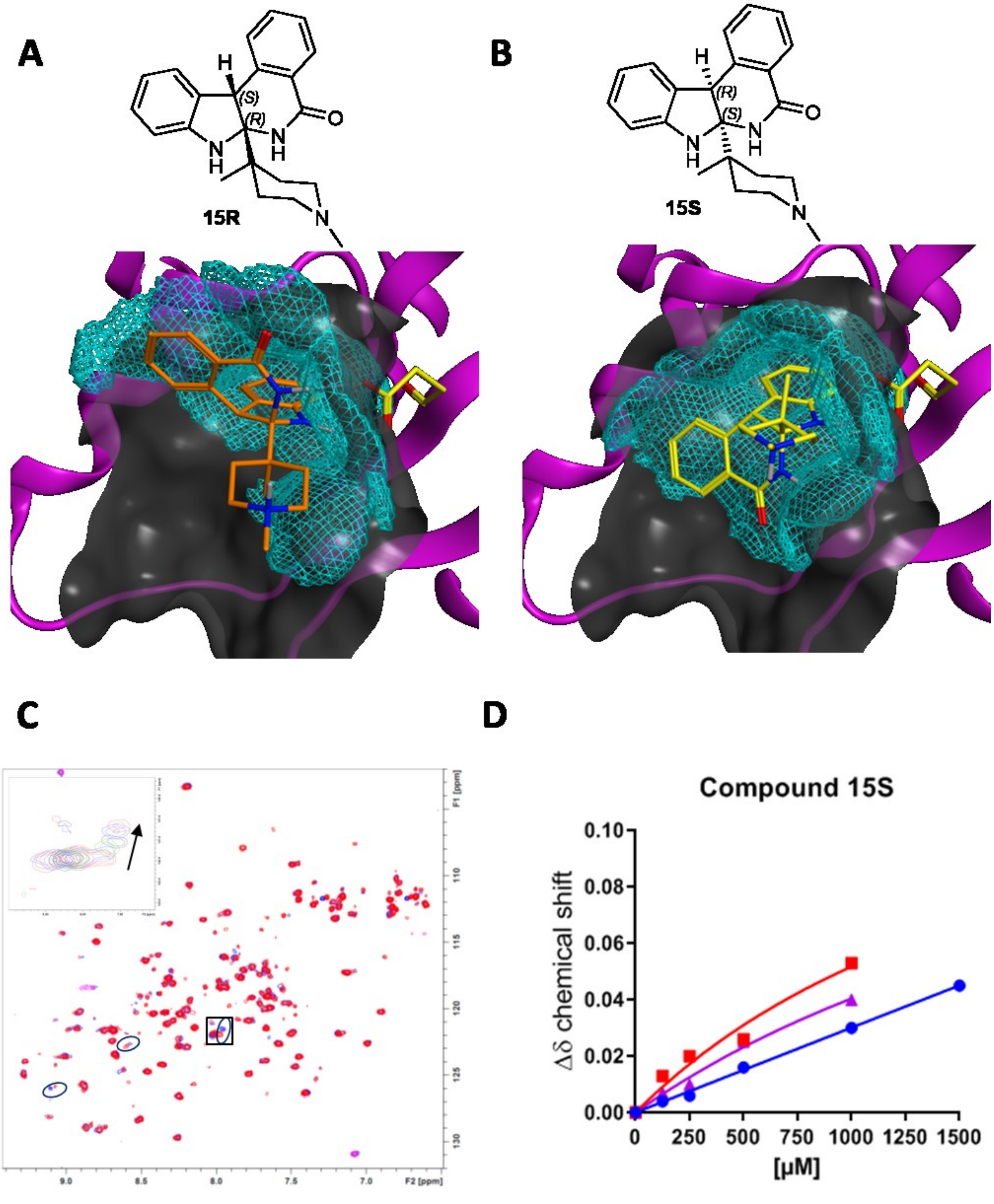

Figure S3 | Docking poses of compound 15R and 15S and NMR of 15S. (a) Chemical structure and snugness of fit iso-contour surface of the docking pose for **15R** (snugness ratio = 0.492, Glide score = -4.623). (b) Chemical structure and snuggness of fit iso-contour surface of the docking pose for **15S** (snugness ratio = 0.568, Glide score = -5.246). (c) Dose dependent cross peak shifts in the 2D ^{1}H/^{15}N HSQC NMR spectra of GCP-KRAS^{G12D} on addition of **15S** (0, 250, 500, 1000 and 1500 µM depicted in the insert, overview shown at 500µM). (b) NMR K_D titration of **15S** binding to GCP-KRAS^{G12D} showing dose dependent shifts without saturation (two peaks exchange broadened at 1.5mM).

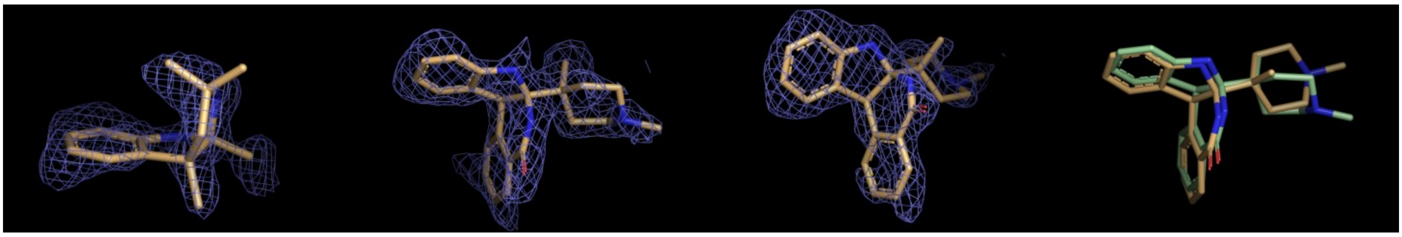

Figure S4 | Electron density of bound compounds. Shown are the $2F_{obs}$-F_{calc} maps (blue grid) for **9b** (panel A), **15** (panel B) and **15R** (panel C) at a cutoff of 0.8 σ. From the racemate of **15**, only the higher affinity diastereomer **15R** could be reliably modelled into the density. The piperidyl-ring in general appears conformationally flexible, and adopts slightly different conformations between the two crystal structures (panel D).

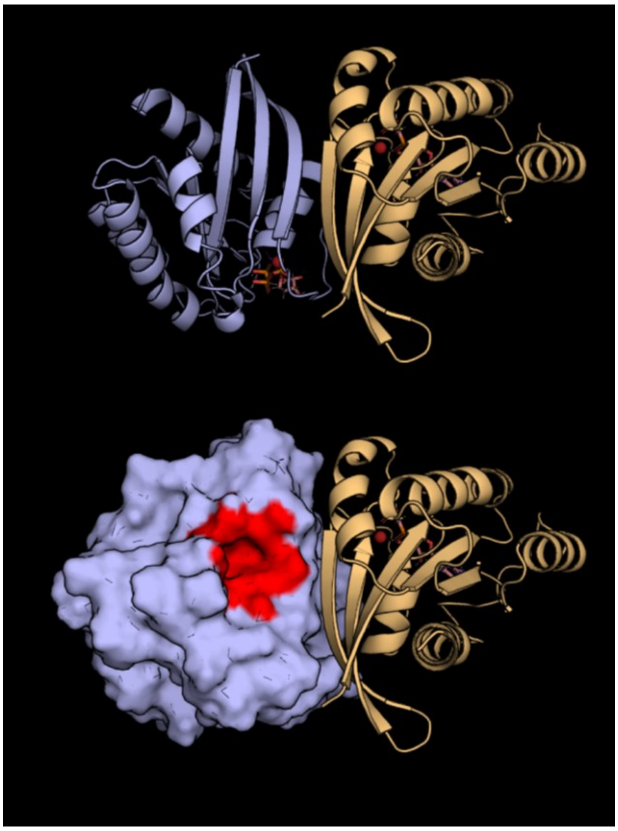

Figure S5 | Structural overview of KRAS and binding site. A) KRAS forms a dimer through its beta-sheet in the structure. Bound GCP is depicted in more detail as sticks. B) Binding site (red) of natural product inhibitors presented in this work.

[1] A. Eberth, M. R. Ahmadian, *Curr Protoc Cell Biol* **2009**, *Chapter 14*, Unit 14 19.
[2] C. Vonrhein, C. Flensburg, P. Keller, A. Sharff, O. Smart, W. Paciorek, T. Womack, G. Bricogne, *Acta Crystallogr D Biol Crystallogr* **2011**, *67*, 293-302.
[3] W. Kabsch, *Acta Crystallogr D Biol Crystallogr* **2010**, *66*, 125-132.
[4] M. D. Winn, C. C. Ballard, K. D. Cowtan, E. J. Dodson, P. Emsley, P. R. Evans, R. M. Keegan, E. B. Krissinel, A. G. Leslie, A. McCoy, S. J. McNicholas, G. N. Murshudov, N. S. Pannu, E. A. Potterton, H. R. Powell, R. J. Read, A. Vagin, K. S. Wilson, *Acta Crystallogr D Biol Crystallogr* **2011**, *67*, 235-242.
[5] I. J. Tickle, Flensburg, C., Keller, P., Paciorek, W., Sharff, A., Vonrhein, C., Bricogne, G., **2018**.
[6] A. J. McCoy, R. W. Grosse-Kunstleve, P. D. Adams, M. D. Winn, L. C. Storoni, R. J. Read, *J Appl Crystallogr* **2007**, *40*, 658-674.
[7] P. D. Adams, P. V. Afonine, G. Bunkoczi, V. B. Chen, I. W. Davis, N. Echols, J. J. Headd, L. W. Hung, G. J. Kapral, R. W. Grosse-Kunstleve, A. J. McCoy, N. W. Moriarty, R. Oeffner, R. J. Read, D. C. Richardson, J. S. Richardson, T. C. Terwilliger, P. H. Zwart, *Acta Crystallogr D Biol Crystallogr* **2010**, *66*, 213-221.
[8] O. S. Smart, T. O. Womack, C. Flensburg, P. Keller, W. Paciorek, A. Sharff, C. Vonrhein, G. Bricogne, *Acta Crystallogr D Biol Crystallogr* **2012**, *68*, 368-380.
[9] P. Emsley, B. Lohkamp, W. G. Scott, K. Cowtan, *Acta Crystallogr D Biol Crystallogr* **2010**, *66*, 486-501.
[10] in *Molecular Operating Environment (MOE), 2013.08*, Chemical Computing Group ULC, 1010 Sherbooke St. West, Suite #910, Montreal, QC, Canada, H3A 2R7, **2015**.
[11] Dassault Systèmes, San Diego, **2015**.
